# Synchronized cell attachment triggered by photo-activatable adhesive ligands allows QCM-based detection of early integrin binding

**DOI:** 10.1038/srep09533

**Published:** 2015-03-31

**Authors:** Jagoba Iturri, Luis García-Fernández, Ute Reuning, Andrés J. García, Aránzazu del Campo, Marcelo J. Salierno

**Affiliations:** 1 Max Planck Institute for Polymer Research, Ackermannweg 10, 55128 Mainz, Germany; 2 Clinical Research Unit, Dept. for Obstetrics & Gynecology, Technische Universitaet München, Munich, Germany; 3 National Scientific and Technical Research Council, Av. Rivadavia 1917, C1033AAJ CABA, Argentina; 4 Woodruff School of Mechanical Engineering and Petit Institute for Bioengineering and Bioscience, Georgia Institute of Technology, Atlanta, GA 30332, USA

## Abstract

The Quartz Crystal Microbalance with dissipation (QCM-D) technique was applied to monitor and quantify integrin-RGD recognition during the early stages of cell adhesion. Using QCM-D crystals modified with a photo-activatable RGD peptide, the time point of presentation of adhesive ligand at the surface of the QCM-D crystal could be accurately controlled. This allowed temporal resolution of early integrin-RGD binding and the subsequent cell spreading process, and their separate detection by QCM-D. The specificity of the integrin-RGD binding event was corroborated by performing the experiments in the presence of soluble cyclicRGD as a competitor, and cytochalasin D as inhibitor of cell spreading. Larger frequency change in the QCM-D signal was observed for cells with larger spread area, and for cells overexpressing integrin α_v_β_3_ upon stable transfection. This strategy enables quantification of integrin activity which, in turn, may allow discrimination among different cell types displaying distinct integrin subtypes and expression levels thereof. On the basis of these findings, we believe the strategy can be extended to other photoactivatable ligands to characterize cell membrane receptors activity, a relevant issue for cancer diagnosis (and prognosis) as other several pathologies.

Integrins are the major adhesive receptors that support cell attachment and migration^1^. The type, density, and affinity of integrins define the adhesive properties of a cell towards the extracellular matrix or a defined biomaterial^2^. The overall integrin activity profile of a cell is in balance with the cellular microenvironment, being altered in many pathological cases^3^,^4^,^5^. Particularly, during cancer progression integrin α_V_β_3_ is overexpressed on many tumor cells^4^,^6^, promoting, for instance, metastasis in breast cancer^7^, reason why this type of integrins were used as a therapeutic target^8^,^9^. Furthermore, priming of integrins by protein Rap1 promotes prostate cancer metastasis^10^, indicating that not only the expression but also the activity of integrins is a key factor in disease development and progression. Understanding integrin expression patterns and priming mechanisms that regulate cell adhesion and migration/invasion, and how they correlate with disease development and progression may provide new diagnostic tools, in particular for cancer.

Quantification of cell adhesion events using acoustic and electric crystal resonators has been reported^11^,^12^,^13^,^14^,^15^,^16^. Using the quartz crystal microbalance with dissipation (QCM-D) method, cell attachment to the crystal resonator is reflected as a decrease in frequency and an increase in dissipation signals, indicating an increase in mass and viscoelasticity of the surface layer^17^,^18^,^19^,^20^,^21^. However, the information obtained by QCM-D studies has so far been of rather limited use, since the QCM-D signal reflects an uncorrelated measure of cell sedimentation, attachment, and spreading occurring at different time scales. These events are not synchronized among the cell population and they cannot be differentiated in the QCM-D curve.

Cell sedimentation takes place within a time scale of seconds to a few minutes depending on cell-substrate interactions^22^,^23^. Cell sedimentation approaches the cell membrane to the surface and enables specific binding of membrane integrins to adhesive ligands at the surface. Subsequently, integrin clustering, focal adhesion (FA) assembly and maturation and cytoskeletal rearrangements occur. These processes can last for several hours. The QCM-D signal reflects the sum of all these processes across the QCM-D sensor surface starting from the time point of cell injection into the QCM-D chamber. We hypothesized that in order to obtain a QCM-D signal characteristic for the integrin binding event, the integrin ligand should be made available to the cells at a defined time point once cell sedimentation has been accomplished. This could generate a time window for detection and quantification of the initial integrin-ligand recognition event, which would reflect integrin expression levels, affinity, and clustering.

Many integrin receptors recognize and bind to the short RGD peptide sequence, and this recognition has been widely exploited to direct drug targeting^8^,^9^,^24^, integrin imaging^25^, and cell adhesion, isolation and migration^26^,^27^,^28^,^29^,^30^,^31^. We have recently developed a photo-activatable variant of the cyclo[RGDfK] cell adhesive peptide, c[RGD(DMNPB)fK] (Figure 1a), that allows light-triggered activation of RGD sites at a surface in the presence of cells^32^. Integrin-RGD-mediated cell attachment, spreading, and migration onto surfaces functionalized with c[RGD(DMNPB)fK] was initiated after a short (seconds) light pulse^33^. In the present study, we used QCM-D crystals modified with c[RGD(DMNPB)fK] functionalized self-assembled monolayers of PEG-thiols in order to monitor integrin binding events during early cell attachment. Due to the protein adsorption-resistant PEG coating, the crystals do not allow non-specific integrin binding to the surface. *In situ* activation of the RGD ligand by light exposure allowed us to precisely define the onset for integrin binding, and a time window to monitor integrin binding separately from membrane spreading. We demonstrate that the QCM-D signal is specifically associated to integrin-RGD binding events and correlates with integrin expression levels. This method allows differentiation among different cell types, and also among cells from the same type displaying different integrin expression levels and compositions. This strategy may open new pathways for sensing and discrimination between pathogenic and healthy cells by reflecting their overall integrin activity level.

## Results

### Sequential detection of integrin binding and spreading on QCM-D crystals functionalized with the photo-activatable RGD peptide after *in situ* light exposure

**Figure 1:**
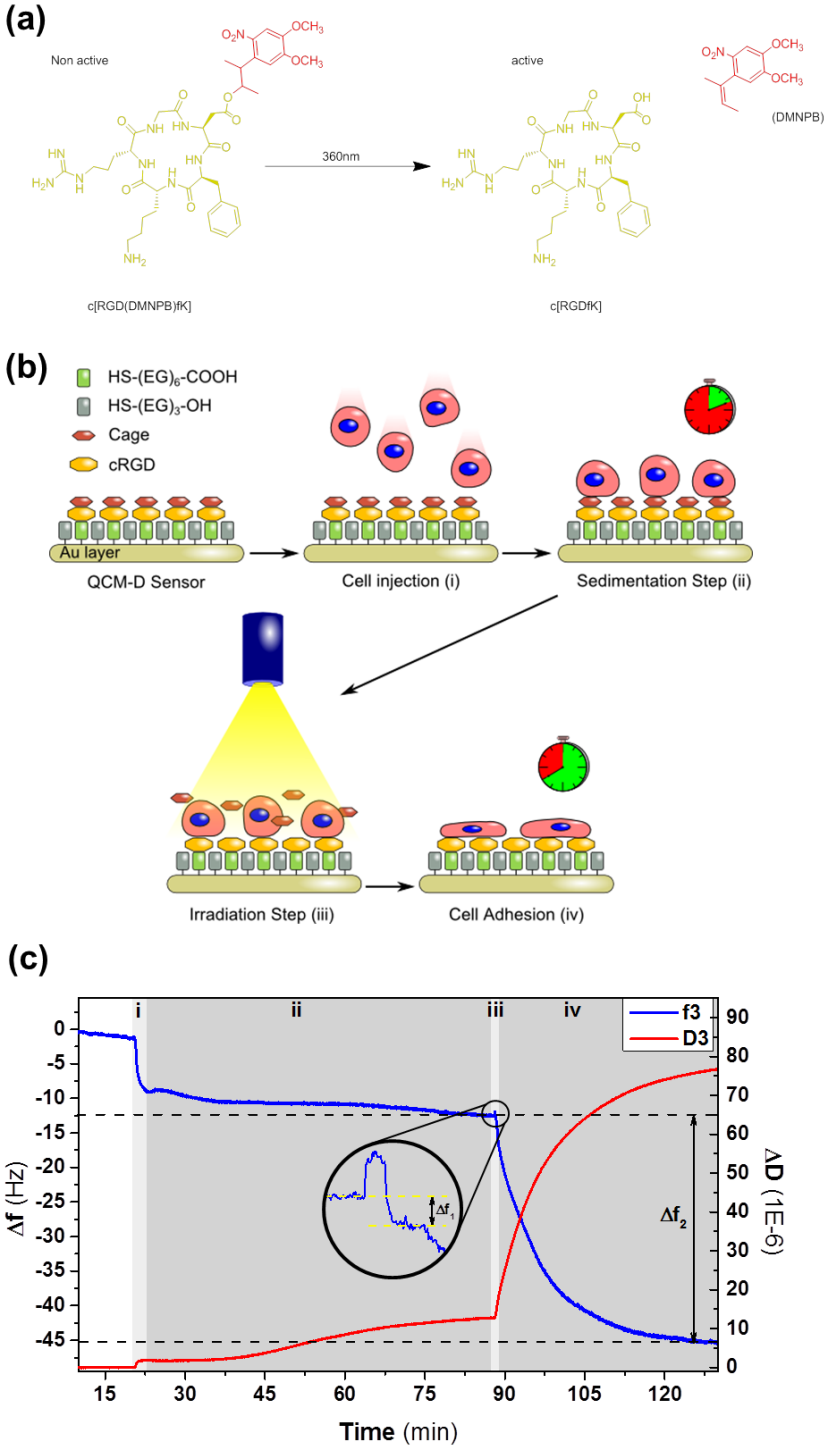
(a) Chemical structure of the photo-activatable adhesive peptide ligand c[RGD(DMNPB)fK] and the products generated upon photolysis. (b) Schematic of the steps to trigger synchronized cell adhesion and spreading. (c) QCM-D measurement of a integrin/RGD-mediated cell binding process on the c[RGD(DMNPB)fK] crystal. Changes in frequency f (blue line) and dissipation D (red line) through the experiment described in Figure 1b. i- cell seeding; ii- cell sedimentation; iii- UV irradiation through the window of the QCM-D chamber for RGD peptide activation (magnified inset); iv- integrin binding and cell spreading.

Gold-coated quartz sensors were functionalized with c[RGD(DMNPB)fK] (Fig. 1a) terminated self-assembled monolayers (SAMs) and inserted into the QCM-D chamber. After equilibration with PBS, the crystals were incubated with a cell suspension (Fig. 1b, i). A slightly decreased frequency and an increased dissipation signal were noticeable, indicating that cells deposited on the crystal but did not strongly interact with it. This is in agreement with the cell repellent properties expected from the pegylated SAM and the inactive cyclo[RGD(DMNPB)fK]. At some point, both frequency and dissipation achieved a constant value, usually after an incubation time of around 40 minutes, indicating that the cell sedimentation on the crystal was completed (Fig. 1b, ii). Similar results have been reported using lipid vesicles^34^,^35^.

RGD peptide activation was performed by illuminating the QCM-D crystal for 30 sec with a LED (λ = 360 nm) through the window of the QCM-D chamber (Fig. 1b, iii). UV light cleaves the DMNPB chromophore and activates the RGD peptide ligand at the sensor surface. At this point, a pronounced drop in frequency and increase in the dissipation signals were visible, indicating an increase in mass and viscoelasticity of the surface layer bound to the crystal. We attribute this increase in mass and viscoelasticity to coordinated recognition and binding of the membrane integrins to the activated RGD ligands on the crystal and subsequent membrane spreading (Fig. 1b, iv)^32^,^33^. The signal reached equilibrium after approx. 2 hours (Fig. 1c, iv). Importantly, frequency and dissipation changes vary with the density of the injected cell suspensions (Supplementary Fig. SI1). The experiments were standardized to a cell concentration of 3 × 10^5^ cells/mL. Under these conditions, cells did not exceed 70% confluence after final spreading on the QCM-D crystal and cell stacking was prevented.

**Figure 2:**
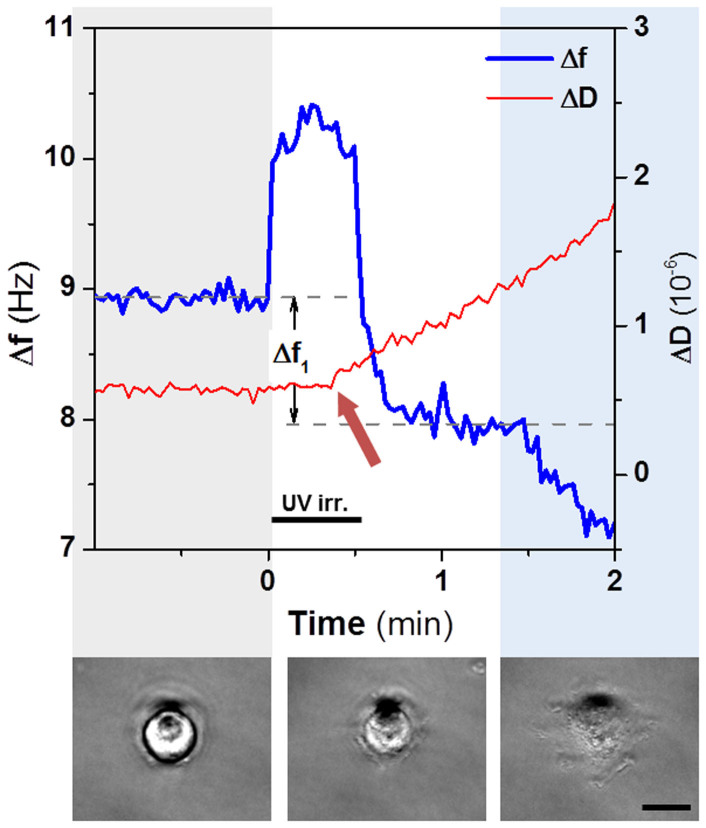
QCM-D signal and cell morphology changes following photo-activation of the RGD ligand. Frequency (blue line) and dissipation (red line) variation shortly before and 2 min after UV light exposure of 30 s from minute 0 (black bar). **Δf_1_** indicates the frequency drop within the first minute after the lamp was turned on. The red arrow points out the moment at which ΔD increase occurs, after 25 s of lamp irradiation. Lower panel: phase contrast images showing the morphology of an adhering cell during the time sequence at 0, 1 and 5 min after irradiation, respectively. Scale bar 10 μm.

Upon UV irradiation (t = 0 s), an immediate ~ 1 Hz jump in the frequency signal was detected (Fig. 2). The frequency jump dropped back down when the UV light was turned off (t = 30 s). The same effect was observed when the same substrates were irradiated in the absence of cells, but the frequency signal returned to the original levels (Supplementary Fig. SI2). We attribute this frequency jump to photo-induced noise, as previously reported^36^,^37^. No corresponding change in the dissipation signal was observed. A ~ 0.5–1.5 Hz decay in the frequency signal also occurred during the first 50 seconds (Fig. 2, Δf_1_), although it only became visible after the photo-induced noise vanished. Simultaneously, the dissipation signal (not affected by the photo-induced noise) showed an increase starting at t = 25 s (red arrow, Fig. 2). We reasoned that these changes were associated with the binding of integrins at the cell membrane to the activated RGD peptide, which occurred rapidly and reached steady state for the frequency signal within 1 min. Parallel live cell microscopy showed that cells flattened during the first 1.5 min after irradiation, and spread at longer times (Supplementary Movie 1), suggesting that the regions iii and iv of the QCM-D curve in figure 1c correspond to integrin binding and the subsequent spreading process separately. These two processes occur at very different time scales (1–2 min *vs* 2 h). Notably, these events have not been observed in previously reported QCM-D measurements of cell-surface interactions using c[RGDfK]^17^,^38^. The possibility of *in situ* activation of the RGD ligand at the surface once cell sedimentation is concluded, allows temporal definition of the onset for integrin-RGD interaction and its detection over the first minute after ligand exposure.

### Specificity of Δf_1_ for integrin binding

**Figure 3:**
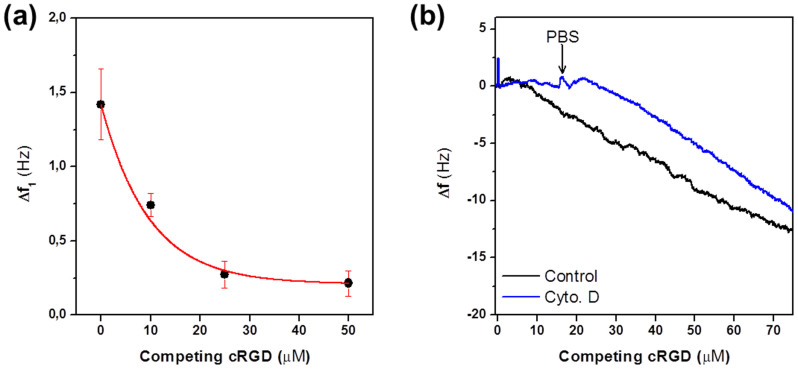
Integrin binding and not spreading is responsible for Δf_1_. (a) Binding experiments were performed in the presence of increasing concentrations (0 to 50 μM) of competing soluble RGDfK peptide ligands resulting in monotonic decrease of Δf_1_. (b) Cells treated with Cyt. D were no longer able to spread after irradiation at minute 1 (blue line), as observed from the unchanged frequency, but were capable of accomplishing firm initial attachment since they remained immobilized on the substrate upon rinsing the chamber with PBS (minute 15). When adding Cyt. D-free PBS again, the cells regained their spreading properties again comparable to control cells (black line). Microscopy images confirmed these results by showing cells in a rounded shape in the presence of Cyt. D, and the usual flattening after a system rinsing with PBS (pictures not shown).

In order to prove that the initial frequency Δf_1_ and associated dissipation changes correspond to light-mediated integrin-RGD-triggered cell adhesive events, we performed competition experiments in the presence of soluble RGD peptide ligand. Soluble RGD competes with surface-bound RGD ligands for integrin binding and hampers cell attachment to RGD-functionalized surfaces. Previous work with HT1080 fibroblast on RGD-modified surfaces demonstrated that adhesion and spreading were compromised when concentrations of soluble RGD between 250 and 500 μM were added to the medium^29^. In our experiments, the frequency and dissipation signals of a cyclo[RGD(DMNPB)fK]-modified crystal incubated with HT1080 cells in the presence of 500 μM soluble RGD did not change after light exposure. This result indicates no binding of the integrins to surface-bound, light-activated RGD peptide ligands due to blocking of the integrins by the high concentration of the soluble RGD. Experiments with lower soluble RGD concentrations showed a significant reduction in the initial Δf_1_. Figure 3a shows Δf_1_ obtained in the presence of 0 to 50 μM of soluble c[RGDfK]. Increasing concentrations of c[RGDfK] led to a pronounced decrease of Δf_1_. This result confirms that the QCM-D signal after light exposure is closely associated to RGD-mediated integrin binding.

A possible contribution of cell spreading to Δf_1_ was ruled out by performing similar experiments in the presence of cytochalasin D (Cyt. D), an inhibitor of actin polymerization that impairs cell spreading in a concentration-dependent manner^22^,^39^,^40^. Addition of 10 μM Cyt. D to the medium did not change Δf_1_ values, confirming that this signal is mainly attributed to integrin binding and not to spreading events. In the presence of Cyt. D, no steep drop in frequency was observed at longer incubation times (20 min) as shown in figure 3b, confirming that cell spreading was the major contribution to the steeper change in frequency. When QCM-D chamber was washed with PBS, a change in frequency similar to control experiments was observed, confirming that cells were able to attach to the substrate when the Cyt. D was removed. These results confirm the capacity of the QCM-D technique in combination with photo-activatable RGD ligands to independently detect and quantify early integrin binding during cell attachment to the QCM-D crystal.

### Δf_1_ correlates with integrin expression levels at the cell membrane

Several pathologies, like cancer, are associated with overexpression of certain integrin subtypes at the cell membrane^6^,^7^. Moreover, integrin inside-out signaling regulates integrin affinity and leads to strong changes in the cell adhesive strength and cellular behavior^41^,^42^. For instance, metastatic malignancy in prostate cancer is upregulated by the inside-out activation of the integrins under Rap1 cytoplasmic expression^10^, proving to be the key factor of migration regardless of the integrin levels. Although integrin expression levels can be analyzed by flow cytometry or immunostaining, the QCM technique has the potential advantage of revealing integrin expression levels and activity in the intact, living cell.

In a first attempt to reveal whether different cell types or a particular integrin expression level may be differentiated by Δf_1_ value, we compared cell binding experiments using primary HUVEC and the cell lines HT1080 and OV-MZ-6, respectively. HUVEC and HT1080 cells are highly responsive to the presence of RGD, as demonstrated in previous reports^33^. In order to elucidate the possibility of measuring different levels of integrin expression, the ovarian cancer cell line OV-MZ-6 with low endogenous integrin α_V_β_3_ levels was studied and compared with OV-MZ-6 cell transfectants exhibiting elevated integrin α_V_β_3_ levels.

**Figure 4:**
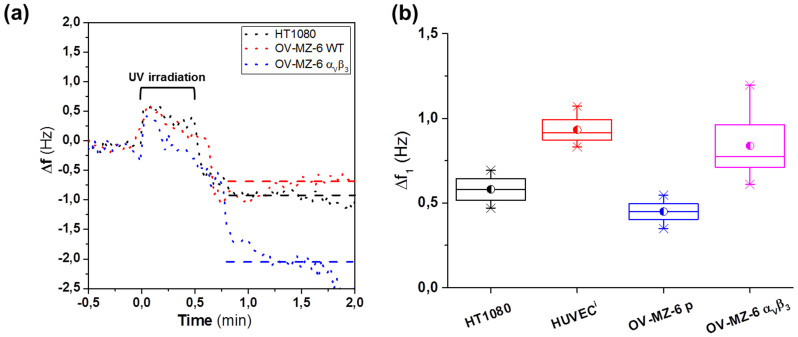
Δf_1_ obtained on different cell types and different integrin expression (a) Changes in frequency produced by RGD activation from minute 0. Δf_1_ was determined by frequency average (striped lines) obtained from 1 minute after irradiation (marked in top) since frequency remains stable for at least that interval. WT and α_v_β_3_ in the figure legend denote the Wild type and α_v_β_3_ transfectant OV-MZ-6 cells. (b) Δf_1_ average obtained for HT1080 (0.618 ± 0.128 Hz), HUVECs (0.966 ± 0.123 Hz) and OV-MZ-6 α_v_β_3_ (0.913 ± 0.245 Hz) and OV-MZ-6 (0.449 ± 0.178) at 3 × 10^5^ cells/ml.

Binding assays were performed as described before. For meaningful comparison, equal cell densities in the incubation solution were used for all cell types. All cell types evaluated showed similar evolution of the QCM-D signals. In all the cases, an initial small frequency drop (Δf_1_) was detected after irradiation (Fig. 4a), then the frequency remains stable for a few seconds (~60 s) before a steeper drop, as a result of cell membrane spreading, started. Figure 4b shows the Δf_1_ drop obtained for each cell type. HUVEC showed significantly higher Δf_1_ than HT1080 cells, most probably due to their larger cell size. The Δf_1_ observed for OV-MZ-6 α_V_β_3_ transfectants was also significantly higher than for the parental cell line (OV-MZ-6 p), as expected due to higher numbers of integrin α_V_β_3_ expression. These results suggest that Δf_1_ is a useful parameter when using photo-activatable RGD to describe the integrin activity of a cell population reflecting expression levels.

We also performed comparative QCM-D experiments using HUVEC as well as OV-MZ-6 cells in the presence of blocking anti-α_V_β_3_ antibodies to abrogate the RGD-integrin recognition event, and also with OV-MZ-6 stably overexpressing integrin α_V_β_3_. HUVEC in the presence of 10 μM anti-α_V_β_3_ antibody showed a 60% decrease in the frequency signal Δf_1_ with respect to the control. The same experiment performed with OV-MZ-6 α_V_β_3_ transfectants gave a 92% decrease in Δf_1_. OV-MZ-6 overexpressing α_V_β_3_ gave a 2-fold increase in Δf_1_ compared to OV-MZ-6 parental cells (Supplementary Table SI1). These results further demonstrate that Δf_1_ can be used as a parameter that reflects integrin expression levels at the cell membrane.

### D/f values reflect cell spreading and focal adhesion maturation

The D/f ratio in the QCM-D signal provides insight into the mechanical properties of the cell membrane attached to the surface^43^,^44^,^45^,^46^. Conceptually, differences in frequency reflect a more direct interaction of the cell with the substrate, while dissipation changes reflect membrane and cytoskeletal rearrangement above the membrane in close contact with the substrate. Previous research provides some experimental evidence for these assumptions^19^,^38^ though the parallel occurrence of cell sedimentation, integrin binding, and cell spreading precludes unambiguous interpretation so far.

**Figure 5:**
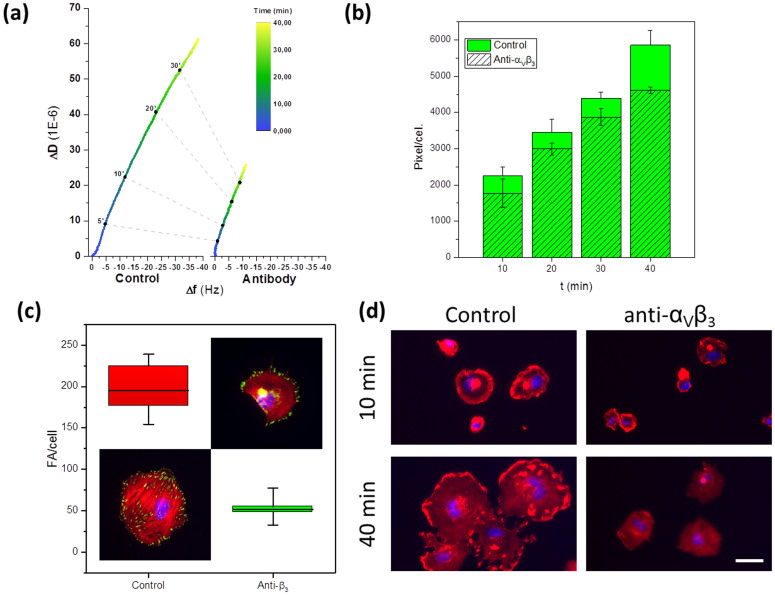
D/f plots modulus and slope may reflect FAs and cell spreading. D/f values of cell spreading (a) with or without 10 μM antibodies directed to integrin α_V_β_3_ on HUVECs. Time progression is indicated with dashed lines. (b) Average of cell spreading progression at different times in the presence or absence of 10 μM antibodies directed to integrin α_V_β_3_. (c) FA per cell after 40 min incubation onto the QCM substrates in the presence or absence of 10 μM antibodies directed to integrin α_V_β_3_. Insets contain representative pictures of HUVEC cells showing vinculin-GFP stained FA in green, DAPI nuclear staining in blue, and TRITC-phalloidin bound to F-actin fibers in red. (d) Representative pictures of cell spreading at 10 and 40 min in the absence (Control) or presence (anti α_V_β_3_) of 10 μM antibodies.

In an attempt to correlate observable parameters of cell adhesion with the D/f plots during spreading, we analyzed the D/f plots of the attachment of HUVEC in presence or absence of anti α_V_β_3_ blocking antibodies (10 μM) (Fig. 5a). In parallel, we analyzed the spreading area and FA formation by fluorescence microscopy. Significantly lower D/f values were obtained in the presence of α_V_β_3_ blocking antibodies, indicating a relationship between D/f at a particular time after exposure and the ability of the cells to adhere and spread. Interestingly, quantification of the area occupied by the cells after spreading (Fig. 5b) showed only ca. 20% lower spreading of the cells treated with the α_V_β_3_ blocking antibodies, while the differences in D/f values were notably larger. The number of FAs was obtained using vinculin-GFP transfected cells in the presence or absence of anti-α_V_β_3_ antibody after 40 min of spreading. A 3.7-fold lower FA density was found in anti-α_V_β_3_ treated cells than in the control (Fig. 5c, d), confirming the unavailability of integrins to cluster in focal contacts. The reduction in the number of FAs seems to correlate better with the observed D/f differences and suggests that the D/f ratio reflects FAs maturation and the associated cytoskeletal changes, more than cell spreading.

## Discussion

QCM-D measurements of integrin-mediated cell attachment on crystals modified with photo-activatable RGD enabled independent characterization and quantification of integrin-mediated cell binding and spreading during the early stages of cell adhesion. The photo-activatable RGD peptide enabled synchronization of the integrin binding event across the cell layer by a short light exposure when sedimentation was completed. This method detected differences in integrin-mediated cell-attachment between different cell types or from same cell phenotype with different integrin expression levels.

Integrin balance is crucial to maintain the integrity of tissue architecture, dictating cell adhesiveness and contributing to cell polarity and functionality by activating specific pathways. For instance, it is already known the imbalance of β_1_ and β_4_ expression in human breast cancer^47^ or the expression of α_v_β_3_ heterodimer seen as a selective cancer stem cell biomarker that leads to therapy resistance^48^. Integrins α_6_ and α_3_ also contribute to cancer initiation being molecular markers of the aggressive cell phenotype^49^,^50^. Differently, activity of β_1_-containing integrins modulates cell adhesion and consequent invasiveness in prostate cancer under higher chemokine levels^51^. In general, increased expression and/or activity of integrins within the primary tumor are associated with poor prognosis and enhanced metastasis in a variety of cancers^52^. A recent review clearly describe the importance of several integrin types involved in the regulation and progression of cancer^53^ establishing that a further understanding is necessary to recognize whether these integrins are interchangeable or specifically required. On this context, identifying cell integrin adhesive changes that influence tumor growing and spreading can lead to discover emerging therapeutic treatments. In this regard, our platform provides information to characterize cell population adhesiveness with the possibility to correlate this with integrin-mediated tumor aggressiveness integrating expression and activity of these receptors. Furthermore, the ability to compare the context effect of integrin adhesion of a normal cellular population with a carcinogenic type can be of great utility to discover specific drugs that reverse this particular condition.

We envision that this strategy could inspire new integrin-based diagnostic methods in pathologies involving changes in membrane integrin expression and avidity. This concept is not only applicable to RGD as recognition motif, but can be extended to other ligands specific for membrane receptors.

## Methods

### Biological reagents

Anti-integrin α_V_β_3_ antibody from Millipore (clone 23C6), cytochalasin D from Sigma (C8273), c[RGDfK] from Peptide International (PCI-3661-PI, M.W. 603.68). Photo-activatable c[RGD(DMNPB)fK] was synthesized as previously reported^32^,^54^.

### Substrate preparation

QSX 301 gold-coated quartz sensors (Q-Sense AB, Västra Frölunda, Sweden) with a fundamental resonance frequency of about 4.95 MHz were incubated overnight in a 1 mM mixed solution of 99% HS(CH_2_)_11_(OCH_2_CH_2_)_3_OH and 1% HS(CH_2_)_11_ (OCH_2_CH_2_)_6_OCH_2_COOH thiols (ProChimia TH 001-m11.n3-0,2 and TH 003-m11.n6-0,1) in absolute ethanol. The substrates were rinsed with ethanol and Milli-Q water and then incubated in an aqueous solution of 0.2 M EDC (N-(3-dimethylaminopropyl)-N′-ethylcarbodiimide hydrochloride), 0.1 M NHS (N-hydroxysuccinimide), 2-(N-morpho)-ethanesulfonic acid (0.1 M) and NaCl (0.5 M)^55^. After 15 min, the solution was removed and the substrates were washed with deionized water and incubated with a solution mix of bovine serum albumin (BSA, Sigma A2153) and c[RGD(DMNPB)fK] in PBS for 1 h in the dark. The addition of BSA was necessary to reduce non-specific attachment of cells to the c[RGD(DMNPB)fK] modified surfaces. The concentration and ratio of BSA and c[RGD(DMNPB)fK] in the mixture was 0.02 mg/ml of c[RGD(DMNPB)fK] and 0.1 mg/ml BSA. Substrates were washed with distilled water and used immediately for cell experiments.

### Cell culture

Human umbilical vein endothelial cells (HUVEC) were grown in M199 basal medium (Sigma, M4530) supplemented with L-glutamine (2 mM), penicillin (1000 U/mL), streptomycin (100 mg/L, Sigma), ECGS supplement (Sigma, E-2759), sodium heparin (Sigma, H-3393) and 20% (v/v) fetal calf serum (FCS) as previously described^56^. HUVEC were used at passages 2 to 6. For cell experiments, cells were treated with 0.25% trypsin and EDTA (1 mM) in Hanks buffer. HT1080 and HeLa cells were cultured in DMEM medium (Invitrogen) supplemented with 10% (v/v) FCS, 1% (w/v) L-glutamine from Invitrogen at 37°C in 5% (v/v) CO_2_. Origin and cultivation of the human ovarian cancer cell line OV-MZ-6^57^ as well as its integrin α_V_β_3_-mediated adhesive, migratory, and proliferative properties were previously described^58^. Stable OV-MZ-6 cell transfectants overexpressing integrin α_V_β_3_ were generated as described earlier^59^.

### QCM-D measurements

QCM-D experiments were performed using a Q-Sense E1 instrument equipped with a window module. The experiments were performed at 37°C in stopped-flow mode. The parameters Δf and ΔD were acquired at six overtones, i = 3, 5, to 13, corresponding to resonance frequencies of f_i_ ≈ 15, 25, to 65 MHz simultaneously at sub-second time resolution. Before being inserted in the chamber, the gold-coated quartz sensors functionalized with cyclo[RGD(DMNPB)fK] terminated self-assembled monolayers were rinsed with EtOH and dried with N_2_ stream. During the experiment, the window of the QCM-D chamber was covered by aluminium foil to avoid photocleavage of the RGD ligand. The QCM-D crystal was equilibrated in PBS. Cells were injected at a concentration of 3 × 10^5^ cells/ml empirically determined to result in maximal signals and avoidance of non-specific cell/surface interactions prior to light irradiation (data not shown). Cells were injected into the chamber at a flow rate of 300 μl/min for 3 min and incubated in stop-flow for ca. 30 min. The QCM-D crystal in the QCM-D chamber was irradiated through the window with a LED (λ = 360 nm, 1.35 mW cm^−2^) for 30 s.

Experiments in the presence of competing soluble cyclic RGD peptides at 10, 25 and 50 μM, cytochalasin D (10 μM) and antibodies rose against integrin α_V_β_3_ (10 μM) were performed by injection of cells in the respective solutions containing these compounds.

### Cell transfection and staining

A vinculin-GFP expression plasmid was used to image vinculin-containing FAs in viable cells. Cells were electroporated using a Lonza transfection kit P5 (Lonza Group Ltd., Basel, Switzerland). At the end of the experiment, cells were fixed with methanol for 5 min and the actin cytoskeleton was stained using TRITC-conjugated phalloidin (Merck-Millipore).

### Microscopical monitoring of cell attachment, spreading and focal adhesion evaluation

Gold coated glass slides functionalized in the same way as QCM-D crystals were incubated with cells and placed in a custom-built set-up stage. Light exposure for activating the RGD peptide was performed with a LED (λ = 360 nm, 1.35 mW cm^−2^) placed on top of the substrate using the microscope light path during 30 seconds. Microscopical images were taken every second by an Oscar digital camera (Allied Vision Technologies, Stadtroda, Germany) mounted on a Leica DM-IRB inverted microscope (Wetzlar, Germany) equipped with a 10 X or 20 X (Leica, NA 0.4). The spreading ratio of the cell was measured in fixed and actin cytoskeleton stained cells using the software Fiji (ImageJ 2.0.0-beta-7.5) to evaluate the number of pixels per cell. For the evaluation of number of FA, fixed cells were immunostained with CD31 antibody (Anti β_3_, Invitrogen). Number of FA was evaluated using the software Fiji (ImageJ 2.0.0-beta-7.5)

## Author Contributions

M.S. and J.I. designed the research. J.I., L.G.F. and M.S. conducted the experiments and the data analysis. A.C., U.R. and A.J.G. supervised the research and contributed with new ideas. U.R. supplied OV-MZ cells. M.S. and A.C. wrote the manuscript. All authors reviewed the manuscript.

## Supplementary Material

Supplementary Information Movie S1

Supplementary Information Supplementary information
