# Supplementary material for: Synchronized cell attachment triggered by photo-activatable adhesive ligands allows QCM-based detection of early integrin binding
Source: Sci Rep. 2015 Mar 31;5:9533. doi: 10.1038/srep09533 (PMC4379501; doi:10.1038/srep09533)
Supplement: Supplementary Information [file srep09533-s2.pdf]

Synchronized cell attachment triggered by photoactivatable adhesive ligands allows QCM-based detection of early integrin binding

Jagoba Iturri[(1)], Luis García-Fernández[(1)], Ute Reuning[(3)], Andrés J. García[(4)], Aránzazu del Campo[(1)]*, Marcelo J. Salierno[(1,2)]*

[(1)] Max Planck Institut for Polymer Research, Ackermannweg 10, 55128 Mainz, Germany.

[(2)] National Scientific and Technical Research Council. Av. Rivadavia 1917, C1033AAJ CABA, Argentina.

[(3)] Clinical Research Unit, Dept. for Obstetrics & Gynecology, Technische Universitaet München, Munich, Germany.

[(4)] Woodruff School of Mechanical Engineering and Petit Institute for Bioengineering and Bioscience, Georgia Institute of Technology, Atlanta, GA 30332, USA

FIGURES

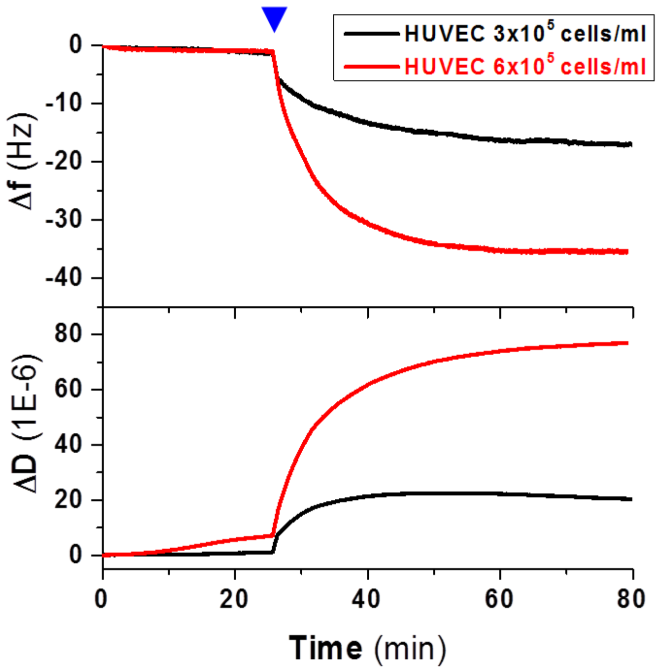

Figure SI1. QCM-D monitoring of HUVEC attachment to RGD(DMNPB)fK crystals using two different cell concentrations. Arrow head shows the onset of UV-LED exposure (i.e. RGD activation). The frequency and dissipation changes associated with cell attachment increase with the density of the injected cell suspensions.

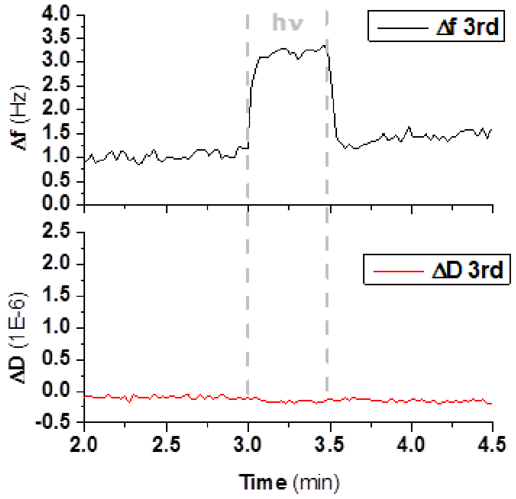

Figure SI2. Photoinduced noise in the QCM curves as consequence of the irradiation of the crystal. A RGD(DMNPB)fK modified crystal was irradiated for 30 s with LED (λ = 405 nm) lamp. The experiment was performed in PBS. When the lamp was turned on (t = 0 s), an immediate jump in the frequency signal was detected. The jump turned back when the LED light was turned off (t = 30 s). The dissipation signal did not show any changes.

TABLES

	Control	Anti $\alpha_v\beta_3$
**HUVEC**	0.97±0.12Hz	0.40±0.15Hz
**OV-MZ-6-wt**	0.45±0.18Hz	--
**OV-MZ-6- $\alpha_v\beta_3$**	0.91±0.24Hz	0.020±0.11Hz

Table SI1. Average value of Δf_1 corresponding to the attachment of HUVEC and OV-MZ-6 cells to RGD(DMNPB)fK functionalized crystals after light exposure in the presence and absence of *anti*-$\alpha_v\beta_3$ antibody. The addition of the antibody significantly reduces integrin binding response. Cell concentration used in all experiments was 3 x 10^5 cells/ml. Data are given as mean ± s.d.

MOVIES

Movie1. Cell adhesion after RGD(DMNPB)fK photoactivation. Cells seeded on Crystals functionalized with RGD(DMNPB)fK were followed by time-lapse microscopy. LED 360 nm irradiation was performed during 30 s in the time interval covered by 0,5 - 1,5 seconds in the movie. Objective used was 40 X magnification. Pictures were taken every 2 seconds and movie frame rate was set at 15 pictures per second.
